# Everybody's Page

**Published:** 1892-05-21

**Authors:** 


### EVERYBODY'S PAGE.

The following delightful announcement appears in a certain foreign inn: "In this hotel the wines leave the traveller nothing to hope for."

---

Equal portions of soft soap and cyanide of potassium mixed with double the quantity of whitening and a little water make a good mixture for removing rust from iron, which, after the operation, should be wiped dry and rubbed over with oil.

---

The practice, which is somewhat too prevalent in the States, and is certainly pregnant with danger to the public, of chemists prescribing as medical practitioners without a licence, has recently received a severe and timely check in one large city, where two chemists were fined 100 dollars for so practising.

---

A foreigner whose command of the English language was not great was much concerned in his mind at the directions of a prescription given him of a powder which was to be taken daily in any "convenient vehicle." He had recourse to the dictionary to help him out of his dilemma, and on finding the definitions of "cart, wagon, carriage, wheelbarrow" applied to the word vehicle, came to the conclusion that his doctor meant him to take carriage exercise and swallow the powder whilst in the vehicle. This he accordingly did, with the most happy result, for in a few weeks the fresh air which he daily took completely cured him of his ailments.

---

How true it is that there is nothing new under the sun is being shown every day. There is after all no novelty in the proposition that the hour and date of the collection of letters should be stamped on them by the Post Office, for this wa actually done 70 years ago and probably discontinued, as Mr. Henniker Heaton suggests, because the department was liable to be convicted by means of it of delaying the delivery of letters. The novelty will lie in a Government department adopting a proposition at once so sensible and reasonable. The despatch with which the Post Office conveys our letters is occasionally remarkable. A few days ago a letter posted in the City at 2 p.m., reached an important suburb not ten miles from town at 10 p.m.

---

Some curious and interesting figures have been published bearing upon the mortality of foundlings in Austria and France respectively. The percentage of deaths in France is surprisingly in excess of those occurring in Austria, but no reason or attempt to account for the discrepancy has, we believe, been put forward.

---

Human beings, especially that portion of them designated by the vague term "society," ever have been and so long as human nature remains what it is, ever will be the slaves of fashion, the votaries of which do not always gain unmixed pleasure from their usually harmless weakness. This is evidenced by the unhappy state into which society has been thrown in St. Petersburg by the death of General Grösser from the results of an injection of "vitalin," a so called remedy for rheumatism, and doubtless every other complaint from which helpless humanity suffers. The general does not seem to have been the only victim of the unfortunate "universal remedy" craze, and the state of mind in which some venturesome experimentalists sre now plunged is not an enviable one.

---

It is such a common practice to flavour soup with celery seeds, that the warning against it given in the *Times* last week is most timely, and should be widely known. The experience of Mr. Frederic Mackarness, who hasissued the note of warning, was a very painful one. The clear soup with which he and his wife began dinner was flavoured with seeds bought in a bottle from a greengrocer. The result of the flavouring is best told in his own words, "About ten minutes afterwards I began to feel quite dizzy, and could hardly swallow the food I was eating. . . My wife became so faint that she asked me to help her up to her room at once. . . At the same time also our sight became blurred, our mouths and throats parched, and we both began to feel cold." Though the doctor, who was immediately summoned, applied drastic remedies without delay, both Mr. and Mrs. Mackarness suffered even during the next day from defective vision, and Mrs. Mackarness' hands were slightly paralysed. An analysis of the so-called celery seeds proved them to be pure henbane, a deadly poison. It is clear that householders should at once give up flavouring their soups with celery seeds and use something for which a spurious and poisonous imitation cannot be substituted.

